# Flavonol Composition and Antioxidant Activity of Onions (*Allium cepa* L.) Based on the Development of New Analytical Ultrasound-Assisted Extraction Methods

**DOI:** 10.3390/antiox10020273

**Published:** 2021-02-10

**Authors:** Ana V. González-de-Peredo, Mercedes Vázquez-Espinosa, Estrella Espada-Bellido, Ceferino Carrera, Marta Ferreiro-González, Gerardo F. Barbero, Miguel Palma

**Affiliations:** Department of Analytical Chemistry, Faculty of Sciences, Agrifood Campus of International Excellence (ceiA3), IVAGRO, University of Cadiz, 11510 Puerto Real, Cadiz, Spain; ana.velascogope@uca.es (A.V.G.-d.-P.); mercedes.vazquez@uca.es (M.V.-E.); estrella.espada@uca.es (E.E.-B.); ceferino.carrera@uca.es (C.C.); marta.ferreiro@uca.es (M.F.-G.); miguel.palma@uca.es (M.P.)

**Keywords:** *Allium cepa* L., antioxidant activity, Box–Behnken, flavonoids, quercetin derivatives, multiresponse optimization, onion, phenolic compounds, UHPLC, ultrasound-assisted extraction

## Abstract

The onion is one of the most cultivated and consumed vegetables, and is a very valuable source of antioxidant substances. Every onion variety is rich in flavonols—mainly quercetin derivatives which makes onions the main dietary source of these compounds. This study intends to develop an ultrasound-assisted extraction method (UAE), an economical, green, and efficient technique, that allows us to determine the quality of onion extracts in terms of flavonol composition and antioxidant activity. For this purpose, an ultrasound-assisted extraction method has been optimized to obtain extracts with a high amount of flavonols, as well as with a high antioxidant activity, not only separately using a Box–Behnken design, but also simultaneously, based on multi-response optimization. Multiple response optimization has not only demonstrated an efficacy level similar to that of the individual ones, but it also represents a considerable reduction in terms of cost, time and work. The optimal conditions for simultaneous extractions were determined as follows: 76.8% methanol as an extraction solvent at pH 2 and 58.5 °C temperature, 85% amplitude, 0.9 s cycle, and 0.2:13 g:mL sample mass/solvent volume ratio. Furthermore, the developed method exhibited a high precision level and great recoveries in a rather short extraction time (2 min). These results, together with the development of a fast and simple UHPLC analysis method, lead us to consider the developed UAE method as a suitable technique for the extraction of bioactive compounds from onion matrices.

## 1. Introduction

Oxidative stress is the disturbance in the production of Reactive Oxygen Species (ROS) relative to the antioxidant defenses. Concerning human health, if an overproduction of ROS takes place, it may damage important biomolecules, such as DNA, proteins, lipids, and carbohydrates, which could give place to a diversity of diseases [1]. In addition to human natural anti-oxidant systems, the consumption of external sources of antioxidants is considered a very important way to prevent some of the diseases caused by oxidative stresses. There is, therefore, a growing trend to improve and enrich the human diet with foods with a natural high content of antioxidant compounds. Of all the natural antioxidant compounds that can be found in our diets, polyphenols stand out as the most important ones [2]. In fact, many research studies have demonstrated that the polyphenols that can be found in vegetables and fruits play an important role in reducing the risk of some degenerative diseases, such as cancer, obesity, cardiovascular diseases, or diabetes [3]. Onions (*Allium cepa* L.), among all such vegetables consumed as a source of polyphenols, stands out because of their high flavonoid content.

The onion (*Allium cepa* L.) is one of the main horticultural crops worldwide [4]. Although traditionally grown and consumed, it should be noted that its production has increased by more than 25% in recent years (FAO) [5]. Such an increment is not just based on its interest as a food condiment because of its characteristic taste and smell but also because of its aforementioned importance as a source of antioxidant compounds [6,7,8]. The current and growing knowledge on these compounds has proven that onion extracts and their isolated bioactive compounds exhibit a large number of biological beneficial effects because of its antioxidant, antiplatelet (decrease platelet aggregation and inhibit thrombus formation), antidiabetic, anticancer, antimicrobial, and anti-inflammatory properties [9,10]. All of these factors support the study of the metabolomic profile of onion bulbs as a matter of the utmost interest.

As mentioned above, onions are particularly rich in flavonoids, amongst other antioxidant compounds, with anthocyanins, and flavonols as the compounds that are most often reported [11]. While anthocyanins are mainly found in red onions, flavonols, and especially quercetin 3,4′-*O*-diglucoside and quercetin 4′-*O*-glucoside can be detected in all types of onions regardless of their white, yellow, or red color [5]. Flavonols, because of their ability to scavenge free radicals, are considered antioxidant models [12] and it is precisely these superior health-promoting properties that make them the main object of this work.

In order to control the quality of these compounds, a rapid and efficient extraction and analysis method is to be developed. Solid–liquid extraction methods are most commonly used for the extraction of bioactive compounds, and specifically for the extraction of phenolic compounds from vegetable matrices. Thus, maceration [13], or magnetic stirring [14], are some of the traditional techniques used for the extraction of phenolic compounds from onion matrices. Such techniques present some disadvantages, like the long extraction times that are required to produce satisfactory yields, which in turn implies a high consumption of solvents and, therefore, an increment of costs. This study intends to find a solution to such problems and, therefore, the effectiveness of ultrasound-assisted extraction (UAE) for the extraction of flavonols from red onion has been evaluated. Ultrasound extraction is based on cavitation, a phenomenon by which cell walls are broken and target compounds are released from their natural matrices [15]. As a consequence of this cavitation phenomenon, a greater dispersion of the solid phase in the liquid is achieved and contact interface is improved, which explains why UAE is often preferred against other traditional methods. This means that greater yields can be obtained in a shorter time using smaller amounts of solvent, with the subsequent economic saving that supports UAE as a more environmentally friendly technique [16]. Consequently, in this research study, UAE has been optimized and employed to obtain onion extracts with a high antioxidant activity because of their high content of flavonols.

Having an efficient extraction technique is not enough to obtain the best results. Conditions are, hence, to be optimized for a fully satisfactory outcome. A number of variables may affect UAE efficiency, such as temperature, time, type of solvent or the concentration of the solvent [17,18]. By using the most suitable conditions for the desired purposes, it is possible to maximize the recovery of the compounds of interest, minimize the extracted adjunct compounds and limit the degradation or alteration of the natural state of the extract [19]. Response surface methodology (RSM) is the most often used method for the development and optimization of extraction processes. RSM is a widely used mathematical and statistical method for modelling and analyzing a process in which the response of interest (dependent variables) is affected by various variables (independent variables). The objective of this method is to optimize the response. There are many types of experimental designs and the use of one or the other will depend on the objectives of the experiment and the number of factors to be investigated. Box–Behnken design (BBD) is one of the most widely used methods when the objective of the design is the optimization, i.e., the study of the best process performance, the interaction effect, and the significance of factors. This type of design is characterized by the measuring of just three levels per factor, which makes of it a less costly design than others with the same number of factors (there are five-level factorial designs). Furthermore, the design points are positioned at the middle of the subareas of the dimension k^−1^. In the case of three factors, for instance, the points are located in the middle of the edges of the experimental domain [20]. Therefore, this design is advantageous because it does not contain any points at the extremes of the cubic region that are prohibitively expensive or impossible to test because of physical constraints in experimentation. In addition to studying the individual influence of each factor on the extraction of flavonols and on the antioxidant activity, the analysis of the simultaneous responses is also of great interest, particularly with regard to cost and time-saving in the laboratory. For this purpose, multi-response optimization (MRO) with desirability functions is usually performed. MRO combined with a desirability function approach has been proven to be a useful statistical tool to determine multi-variable problems and to optimize either one or several responses [21].

Therefore, the aim of the present study is the development and optimization of an ultrasound-assisted extraction method to obtain onion extracts with a large flavonol content and a high antioxidant activity. Having efficient extraction methods is very useful because it allows evaluating the quality of the different onion samples. Such a method should allow the selection of those varieties that are richer in bioactive compounds with antioxidant activity and, therefore, with better biofunctional characteristics with regard to health benefits for consumers.

## 2. Materials and Methods

### 2.1. Biological Materials

Red onion samples (purple variety) purchased at a local market in the province of Cadiz, Spain were the materials used for this study. Specifically, the flavonol content and the antioxidant activity of the bulbs were studied. For this purpose, the outer layers of the bulbs were removed and their core was cut into small pieces. The chopped onions were lyophilized in a LYOALFA freeze dryer (Azbil Telstar Technologies, Terrasa, Barcelona, Spain) and crushed by means of a ZM200 knife mill (Retsch GmbH, Haan, Germany), which offers a final fineness <40 µm. Finally, the samples were stored in a freezer at −20 °C until further analysis.

### 2.2. Chemicals and Solvents

Mixtures of methanol (Fischer Chemical, Loughborough, United Kingdom) of HPLC purity and Milli-Q water, obtained from a Milli-Q water purification system (Millipore, Bedford, MA, USA) were used as the extraction solvents. An HCl solution (1 M) and a NaOH solution (1 M) (Panreac, Barcelona, Spain) was used to even pH values. For the analysis of the extracted compounds, the following solvents were employed: methanol and acetonitrile, both HPLC grade and obtained from Panreac (Barcelona, Spain), Milli-Q water, obtained from the aforementioned Millipore purification system, and acetic acid (Merck KGaA, Darmstadt, Germany). Quercetin 3-*O*-glucoside obtained from Sigma-Aldrich (Steinheim, Germany), was used as the commercial standard for the quantification of the extracted compounds. For the study of the antioxidant activity, DPPH (2,2-diphenyl-1-picrylhydrazyl) radical scavenging and Tris base were purchased from Sigma-Aldrich (San Luis, Missouri, USA) and 6-hydroxy-2,5,7,8-tetramethylchroman-2-carboxylic acid (Trolox) (Sigma-Aldrich, Steinheim, Germany) was used as the standard control for antioxidant activity.

### 2.3. Ultrasound-Assisted Extraction

#### 2.3.1. Ultrasound-Assisted Extraction Equipment

To carry out the ultrasound-assisted extraction, a Sonopuls HD 2070.2 processor (BANDELIN electronic GmbH & Co KG, Heinrichstrabe, Berlin, Germany) was used to control the cycle, the amplitude, and the working time. This UAE equipment employed 70 W of maximum output power and a working frequency of 20 kHz. The probe employed was a VS 70 T (BANDELIN electronic GmbH & Co KG, Heinrichstrabe, Berlin, Germany) with the following characteristics: volume between 20–900 mL, diameter 13 mm, length approximately 130 mm, and amplitude 97 µm. An adjustable double vessel thermostatic bath was also used (Frigiterm-10, Selecta, Barcelona, Spain) to maintain the samples at the selected temperature.

#### 2.3.2. Ultrasound-Assisted Extraction Procedure

A series of experiments were carried out to develop the optimized UAE method. For each experiment, approximately 0.2 g of sample was weighed into a Falcon tube and the corresponding solvent volume was added according to each experiment’s sample-solvent ratio. The UAE conditions set for the extractions were: solvent composition (50–100%), temperature (10–60 °C), amplitude (30–90% of the maximum power (70 W)), cycle (0.4–1 s), pH (2–7) and sample–solvent ratio (0.2:10–0.2:20 g sample:mL solvent). The Falcon tube was placed into the double vessel to maintain the sample at the desired temperature, and the ultrasound probe was submerged in it. The initial extraction time was 10 min, followed by a sample cooling time, and the extraction conditions (i.e., cycle, amplitude, and temperature) were set according to each experiment. The extracts obtained were centrifuged and the supernatant was transferred to a 25 mL volumetric flask. The precipitate obtained from the centrifugation was re-dissolved in 5 mL of the same extraction solvent and centrifuged again under the same conditions. The new supernatant was transferred back into the volumetric flask and it was filled up with the same solvent. Finally, the flask content was stored at −20 °C until further analysis.

### 2.4. Flavonols Identification

The extracts were analyzed to detect and determine their content in the compounds of interest. First of all, the flavonols were identified by means of ultra-high performance liquid chromatography (UHPLC) coupled to quadrupole-time-of-flight mass spectrometry (Q-ToF-MS) (Xevo G2 QToF, Waters Corp., Milford, MA, USA). The chromatographic separation was performed in a reverse-phase C18 analytical column (ACQUITY UPLC CSH C18, 100 mm × 2.1 mm i.d., 1.7 mm particle size) with a binary solvent system at a flow rate of 0.4 mL min^−1^. Phase A was 2% formic acid–water solution and phase B was 2% formic acid–methanol solution. Specifically, a rapid UHPLC-Q-ToF-MS method, previously described in the bibliography [22], was employed. Previous to their identification, all the UAE extracts were filtered through a 0.20 µm nylon syringe filter (Membrane Solutions, Dallas, TX, USA). The volume injected was 3 µL. The compounds were individually identified based on their retention time and molecular weight: quercetin 3,7,4′-*O*-triglucoside (2.9 min, *m/z* = 787.1421), quercetin 7,4′-*O*-diglucoside (4.5 min, *m/z* = 625.1396), quercetin 3,4′-*O*-diglucoside (5.138 min, *m/z* = 625.1398), isorhamnetin 3,4′-*O*-diglucoside (5.2 min, *m/z* = 639.1559), quercetin 3-*O*-glucoside (5.3 min, *m/z* = 463.0886), quercetin 4′-*O*-glucoside (5.5 min, *m/z* = 463.0873), and isorhamnetin 4-*O’*-glucoside (5.5 min, *m/z* = 477.1040).

### 2.5. Flavonols Quantification

**Figure 1:**
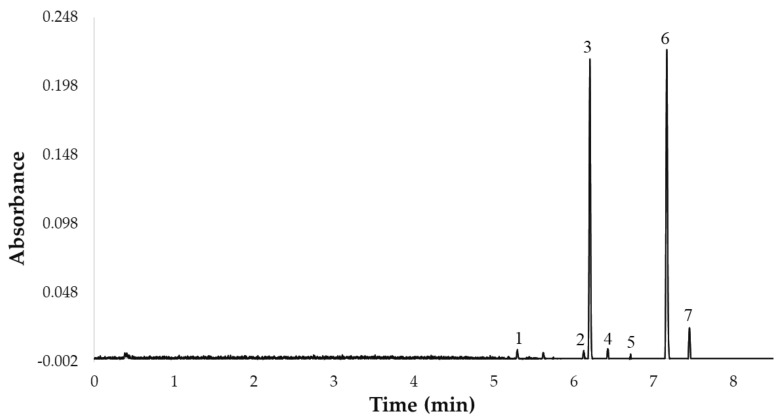
UHPLC chromatogram of the 7 flavonols identified in the UAE extracts from red onion Bulbs at 360 nm: 1. quercetin 3,7,4′-*O*-triglucoside, 2. quercetin 7,4′-*O*-diglucoside, 3. quercetin 3,4′-*O*-diglucoside, 4. isorhamnetin 3,4′-*O*-diglucoside, 5. quercetin-*O*-glucoside, 6. quercetin 4′-*O*-glucoside, 7. isorhamnetin 4′-*O*-glucoside.

Once the 7 flavonols were identified, they were quantified by ultra-high performance liquid chromatography (ACQUITY UPLC^®^ H-Class, Waters Corporation, Milford, MA, USA) coupled to a photodiode array (PDA) detector (ACQUITY UPLC^®^, Waters Corporation, Milford, MA, USA). Specifically, a rapid UHPLC-PDA method with a C18 reverse-phase column (UPLC^®^ BEH C18, Waters Corporation, Milford, MA, USA, 50 mm × 2.1 mm i.d., 1.7 mm particle size), previously published by our team, was employed [23]. The gradient of the UHPLC-PDA method used in this study was as follows: 0.0 min, 0% B; 1.0 min, 0% B; 4.0 min, 5% B; 6.0 min, 10% B; 7.0 min, 20% B; 7.2 min, 20% B; 7.5 min, 40% B; 8.0 min, 45% B; 8.5 min 50% B; 9.0 min, 100% B; 12.0 min, 100% B; 13.0 min, 0% B. The column temperature was 47 °C, the flow rate was 0.6 mL min^−1^, the injection volume was 3.0 µL, and the mobile phase was a binary solvent system. Phase A was 2% acetic acid in water and phase B was 2% acetic acid in acetonitrile. The total analysis time (sample-to-sample) was 13.0 min, including the return to the initial conditions and the re-equilibration of the column, while the separation of the 7 major compounds was completed in less than 8 min. Prior to chromatographic analysis, the extracts were filtered through a 0.20 µm nylon syringe filter (Membrane Solutions, Dallas, TX, USA). The UHPLC chromatogram representing the 7 flavonols is shown in Figure 1.

Once the flavonols were analyzed, they were quantified by means of the calibration curve of quercetin 3-O-glucoside elaborated using 6 points (0.1–200 mg L^−1^) in triplicate. The regression equation (y = 8610.36x + 8282.83) and the determination coefficient (R^2^ = 0.9997) were calculated using Microsoft Office Excel 2013. Each one of the other 6 flavonols was quantified based on the quercetin 3-*O*-glucoside curve, according to the molecular mass ratio of each corresponding compound and assuming that the 7 flavonols have similar absorbance since they have similar chemical structures. All the analyses were carried out in duplicate, and the results were expressed as milligrams of flavonol per g of dry weight sample (DW) (mg flavonols g^−1^ DW).

### 2.6. Antioxidant Activity

The antioxidant activities of polyphenolic mixtures are usually evaluated using different in vitro spectrophotometric-based assays [24]. In this work, the antioxidant activity of the onion extracts was evaluated using DPPH assays according to the procedure designed by Brand-Williams et al. [25] and modified by Miliauskas et al. [26] The molecule of α-diphenyl-*β*-picrylhydrazyl (DPPH; C_18_H_12_N_5_O_6_) is characterized as a stable free radical by the delocalization of the spare electron over the molecule as a whole, so that the molecules do not dimerize, as would be the case with most other free radicals [27]. Due to this delocalization, the DPPH radical exhibits a violet color in the solution because of a strong absorption band at about 515 nm. On mixing the DPPH solution with a substance that can donate a hydrogen atom (antioxidant substances), the odd electron of nitrogen atoms in DPPH is reduced. This phenomenon allows to determine the antioxidant activity of a sample according to how its optical absorption changes at 515 nm with the appearance of a pale yellow color. For this assay, a 6 × 10^−5^ M DPPH solution was first prepared in methanol. Then, for each 100 µL of onion extract, 900 µL and of 0.1 M Tris-HCl buffer (pH 7.4) and 2 mL of the DPPH solution were added to the mixture. The mixture was incubated for 40 min in the absence of light and at room temperature. Then, the absorbance was measured at 515 nm. The linear regression for the trolox standards was constructed using 6 points (0–1.4 mM) in triplicate. The regression equation (y = 88.94x + 0.75) and the determination coefficient (R^2^ = 0.9959) were calculated using Microsoft Office Excel 2013. The results were expressed as mg of Trolox equivalents (TE) per g of dry weight sample (mg TE g^−1^ DW).

### 2.7. Applying a Box-Behnken Design (BBD) to UAE Optimization

For the development and optimization of the UAE method, a response surface Box–Behnken Design (BBD) was carried out. This type of design is characterized by the use of only three levels per factor: a lower level (−1), an intermediate level (0), and a higher level (1). However, the feature that differentiates this design from others is that, in addition to not containing an embedded factorial or fractional factorial design, it does not present axial points, but rather a more spherical arrangement of the design points, which allows to exclude any experiment under extreme conditions [28]. In this work, the following 6 independent factors have been studied: composition of the solvent (% methanol in water) (X_1_), pH of the solvent (X_2_), extraction temperature (X_3_), ultrasound amplitude (X_4_), ultrasound cycle (X_5_), and ratio between the sample mass and solvent volume (“ratio”) (X_6_). The specific values studied for each factor were: solvent composition (50, 75 and 100%), temperature (10, 35 and 60 °C), amplitude (30, 60 and 90%), cycle (0.4, 0.7 and 1 s), pH (2, 4.5 and 7) and sample-solvent ratio (0.2:10, 0.2:15 and 0.2:20 g sample:mL solvent). With respect to the response variables, two responses were studied. The first response was the total concentration of flavonols in the red onion bulbs (Y_TF_, mg g^−1^), calculated as the total sum of the concentrations of each one of the 7 individual flavonols quantified by UHPLC. Since onion extracts are rich in phenolic compounds, mainly comprising quercetin derivatives as well as other bioactive compounds, they are expected to exhibit a good antioxidant capacity. Consequently, their antioxidant activity (Y_DPPH_, mg g^−1^) expressed as mg TE per gram of onion sample was established as the second response variable. Then, according to the abovementioned six independent factors and by applying its specific BBD equation, a design comprising 54 treatments, including 6 repetitions at their centerpoint for calculating the error, was obtained. All experiments were carried out randomly. These experiments and their results can be seen in Table 1.

By treating the results obtained using RSM, a mathematical model is obtained that adjusts as best as possible to the experimental responses obtained based on the conditions used. In this way, a second-order polynomial equation (Equation (1)) was achieved, where the response of the system is expressed as a function of the factors involved as well as of the interactions between those factors.

(1) $$Y=\;\beta_{0}+\sum_{i=1}^{k}\beta_{i}X_{i}+\;\beta_{ii\;}X_{i}^{2}+\;\sum_{i}\sum_{i=1}^{k}\beta_{ij}X_{i}X_{j}+r,$$

In this Equation, *Y* represents the aforementioned responses (*Y_TF_* and *Y_DPPH_*); *β_0_* the model constant; *βi* the coefficient for each main effect; *βij* the coefficient corresponding to the interactions between factor *i* and factor *j*; *βii* the coefficient of the quadratic factors that represent the curvature of the surface; *X* each one of the factors studied; and *r* the residual value (random error). The statistical significance of the model and the regression terms were evaluated by means of an Analysis of Variance (ANOVA). The fit quality of the polynomial model was evaluated using a coefficient of determination (R^2^). Statgraphics Centurion version XVI software (Warrenton, VA, USA) and Design Expert software (Version 13, Stat-Ease Inc., Minneapolis, MN, USA) were used to carry out the experimental design, data analysis, and model construction.

In order to find the optimum extraction conditions for both responses (flavonol content and antioxidant activity) at the same time, an MRO was performed using the desirability functions. For this purpose, the data were first analyzed to generate a separate model for each response. Then, the predicted values that were obtained from each response surface (*Yi)* were converted into individual desirability functions, *di(Yi).* These desirability functions *di(Yi)* are expressed as values between 0 and 1, where *di(Yi)* = 0 represents a completely undesirable value of *Yi* and *di(Yi)* = 1 represents a completely desirable or ideal response value [29]. The geometric mean of the individual desirability values are calculated to obtain the overall desirability D (Equation (2)):

(2) D=d1 × d2 ×…dm1m,

where *di* indicates the desirability of each response and m is the number of responses included in the calculation (in this case, *m* = 2). Finally, in order to compare the results obtained from the independent and the multiresponse methods, a *t*-test was used. For this purpose, normal distribution and the same variance was assumed and an independent two-tailed *t*-test was carried out. *p*-values from the *t*-test that are lower than 0.05 would be considered as significant. All the calculations were performed using Microsoft Office Professional Plus 2016, employing the “T.N.” function.

## 3. Results and Discussion

### 3.1. Stability Study

**Figure 2:**
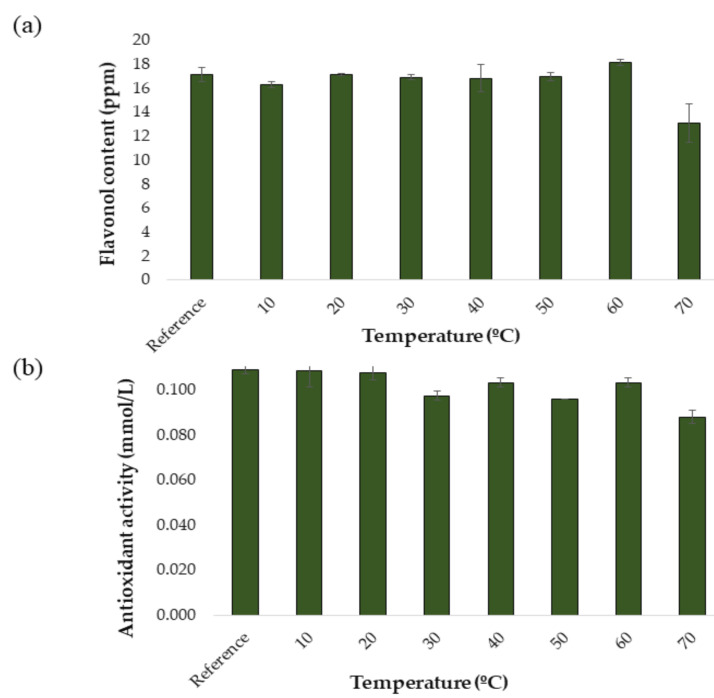
Stability study at different temperatures (*n* = 3): (**a**) flavonols in red onion extracts and (**b**) antioxidant activity in red onion extracts.

For the development and optimization of the UAE method, a response surface Box–Behnken Design (BBD) was carried out. This type of design is cha. Before the UAE method was developed to produce onion extracts from onion samples, a study was carried out to determine the stability of the compounds of interest at different extraction temperatures. This factor is essential for this study, since the temperature is generally a factor that greatly affects extraction performance. In fact, exceedingly high temperatures may lead to the degradation of phenolic compounds, while too low temperatures may not produce the necessary effect in terms of extraction performance [16,30]. For this reason, the effect of varying temperature levels on the extraction performance of a specific protocol while the rest of the conditions remained invariable was to be studied. Thus, the conditions for the temperature tests were set as follows: 50:50 MeOH:H_2_O extraction solvent, 60% ultrasound amplitude, 0.5 s cycle, 15 mL solvent and 20 min extraction time. The same protocol as explained in Section 2.3.2 was applied and the following temperature levels were tested: 10, 20, 30, 40, 50, 60, and 70 °C. All the analyzes were performed in triplicate. To obtain a control extract, an extraction was carried out under the same intermediate conditions but without applying an external temperature. The results obtained are shown in Figure 2a and Figure 2b. In Figure 2a it can be seen how the total content of flavonols varies as a function of the applied extraction temperature. As a result of this study, it was decided to work in a temperature range of 10 to 60 °C, since a decrease in the concentration of flavonols began to be observed in temperatures over 60 °C, probably due to the aforementioned degradation. It can be seen in Figure 2b how the antioxidant activities of the onion extracts vary as a function of the applied extraction temperature. In the case of dpph, the trend shows some ups and downs. However, it can be observed that from 10 °C the anti-oxidant capacity of the extracts is almost constant until reaching over 60 °C. Actually, a drop in the concentration of flavonols, which are responsible for the antioxidant capacity of the extracts, can be observed at 70 °C, which in turn would explain this lower antioxidant activity at temperatures over 60 °C.

### 3.2. Development of the Individual UAE Method for Total Flavonols

#### 3.2.1. Box–Behnken Design for Individual Total Flavonols

After elaborating the matrix from the experiments (Table 1) through the BBD, the analysis of variance (ANOVA) was applied to the set of results in order to evaluate the effect of the factors of interest as well as that of their possible interactions. The results from the ANOVA are shown in Table 2.

Based on test-*F* (model *F*-value of 16.23), the *p*-value of the model was less than 0.05, which means a model significance at the 95% confidence level. However, the lack-of-fit test showed a *p*-value < 0.0001, which indicates evidence for a lack-of-fit, i.e., regression seems inadequate. This value of lack-of-fit is expected due to the nature of the phenolic compounds. The variability between the nature of the bioactive compounds, especially the wide range of polarity, makes that in this type of designs the lack-of-fit test usually appears in this range. In any case, the optimal conditions obtained are a compromise situation to extract the most desirable quantity of these compounds [31]. Furthermore, based on these results, it can be confirmed that the analysis explains 85.01% of the total variability, which is consistent with the statistical similarity between the experimental and predicted values.

In addition to knowing the validity of the obtained polynomial model, the ANOVA indicates the coefficients for the different parameters of the quadratic polynomial equation and their significance (*p*-values). Furthermore, it is also possible to determine which factors and/or interactions have a relevant influence on the response. Those factors and/or interactions with *p*-values lower than 0.05 were considered to have a relevant influence on the response at the established level of significance (95%).

**Figure 3:**
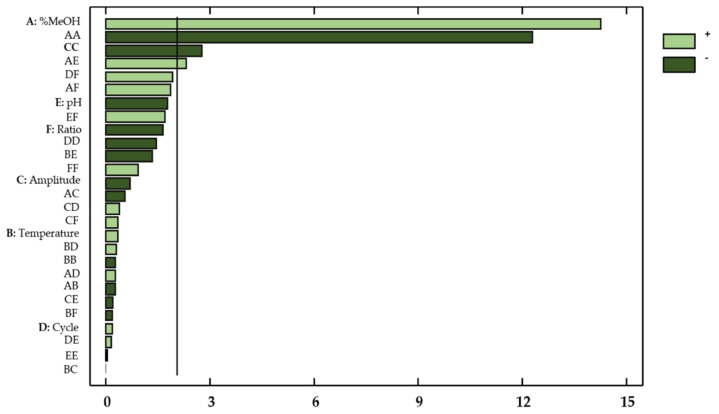
Standardized Pareto Chart for total flavonols in onion extracts. Light green (positive effect); Dark green (negative effect).

This information was supplemented by a Pareto chart (Figure 3), where the influencing factors or interactions can be easily observed.

With regard to the concentration of flavonols in onion extracts, only the linear variable percentage of methanol in the solvent (X_1_) showed a significant effect on the flavonol extraction yields (*p*-value < 0.05). Regarding the possible influences from factor interactions, the quadratic interaction percentage methanol–percentage methanol (X_1_^2^), the quadratic interaction amplitude–amplitude (X_3_^2^), and the percentage methanol–pH interaction (X_1_X_5_) also showed a relevant effect on the response. These results are consistent with those reported in the literature, which indicates that the percentage of methanol, pH, and amplitude are highly influential variables on the extraction of bioactive compounds from natural matrices [32,33,34].

Specifically, the percentage of methanol showed a positive effect on the response variable (b_1_ = 1.48), which indicates that an increase in the percentage of methanol in the solvent favors the extraction of flavonols from red onion. It is also known that hydroalcoholic mixtures are more efficient than pure solvents for the extraction of amphiphilic or moderately polar molecules, such as polyphenols [33]. This is due to the intermediate polarity of such mixtures, which is similar to the phenolic compounds. This similar polarity enhances the solubility of the compounds of interest into the solvent, which in turn favors their subsequent extraction.

A second-order mathematical model from the coefficients of the effects and interactions (Table 2) can be obtained that would allow us to predict the Y_TF_ response values as a function of the independent variables (Equation (3)):

*Y_TF_*(mg g^−1^) = 7.50 + 1.48·*X_1_* + 0.036·*X_2_* − 0.072·*X_3_* + 0.019·*X_4_* − 0.19·*X_5_* − 0.17·*X_6_* − 1.94·*X_1_^2^* − 0.050·*X_1_X_2_* − 0.099·*X_1_X_3_* + 0.037·*X_1_X_4_* + 0.41·*X_1_X_5_* + 0.34·*X_1_X_6_* − 0.046·*X_2_^2^* + 0.00057·*X_2_X_3_* + 0.053·*X_2_X_4_* − 0.17·*X_2_X_5_* − 0.035·*X_2_X_6_* − 0.44·*X_3_^2^* + 0.070·*X_3_X_4_* − 0.038·*X_3_X_5_* + 0.045·*X_3_X_6_* − 0.23·*X_4_^2^* + 0.031·*X_4_X_5_* + 0.34·*X_4_X_6_* − 0.0082·*X_5_^2^* + 0.31·*X_5_X_6_* − 0.15·*X_6_^2^*, (3)

This mathematical model can be reduced by omitting the irrelevant terms (*p*-value > 0.05). The reduced equation (Equation (4)) for total flavonols was expressed as follows:

*Y_TF_* (mg g^−1^) = 7.50 + 1.48·*X_1_*− 1.94·*X_1_^2^* + 0.41·*X_1_X_5_* − 0.44·*X_3_^2^*, (4)

**Figure 4:**
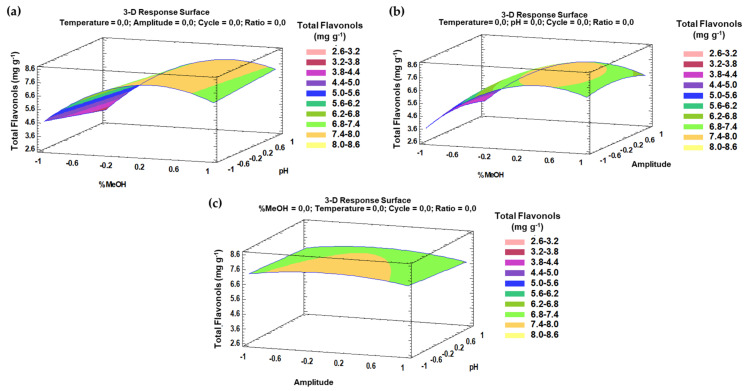
3D surface plots of the Box–Behnken design using polynomial equations: (**a**) effect from solvent composition and pH on the total flavonol extraction; (**b**) effect from solvent composition and amplitude on the total flavonol extraction; (**c**) effect from amplitude and pH on the total flavonol extraction.

The trends that have been outlined above were recorded in three-dimensional (3D) surface plots using the fitted model to better understand the main effects and the interaction effects from the most influential parameters. The combined effects of methanol-pH, methanol–amplitude, and amplitude–pH on the total flavonol recovery are represented in Figure 4a–c.

#### 3.2.2. Optimal Conditions for the Individual Extraction of Total Flavonols

The optimal values of each factor to obtain the maximum response, that is, the best possible extraction of flavonols, was determined based on the Box–Behnken design. Thus, the following UAE optimal values were obtained: 79% methanol in water extraction solvent with pH 2, 60 °C extraction temperature, 53.5% ultrasound amplitude, 0.54 s ultrasound cycle, and 0.2 g:10.8 mL sample–solvent ratio. The optimal temperature obtained was 60 °C, which corresponds to the upper end of the studied interval. However, it was decided not to carry out experiments at higher temperatures because of the degradation of flavonols that had been previously observed in the stability study (Section 3.1). This high temperature agrees with other results found in the literature according to which high temperatures increase the mass transfer of the molecules of interest and the solubility, resulting in a higher overall yield [35,36,37]. The optimal percentage of methanol is also high (79%), which indicates that the extracted flavonols are moderately polar and therefore are better dissolved into solvents containing a higher percentage of methanol than water. An acid value of pH was also found to be the optimal value since this increases the efficiency of the extraction, as it plays a key role in the breaking down of the cell membranes that release the flavonols for their solubilization [38,39]. Regarding the amplitude, its optimal level was established at an intermediate value (53.5%). Since a certain amount of energy is required to release the target compounds from the matrix, an excess of energy may also speed up the degradation process of the phenolic compounds [16]. Thus, it has been observed that during ultrasound-assisted extraction (for 30 min) it can produce degradation of up to 75% [40] and all reactions are promoted when high amplitudes are used [41], including the formation of free radicals. In these cases, flavonols can act as scavenging compounds in reactive oxygen species and then undergo oxidation reactions.

#### 3.2.3. Optimal Time and Precision for the Extraction of Individual Total Flavonols

**Figure 5:**
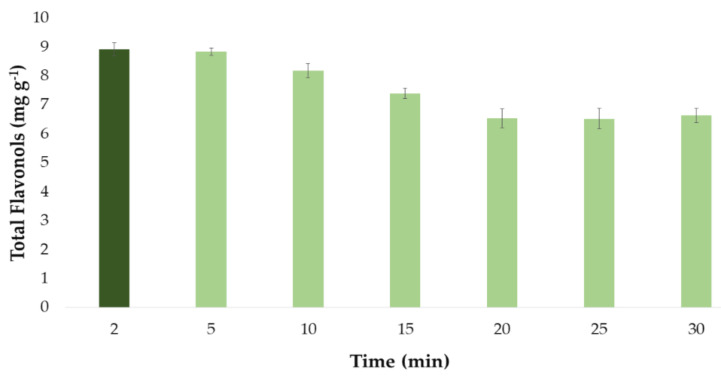
Optimal time (*n* = 3) for the extraction of total flavonols from onion extract according to the developed UAE method.

Once the optimal values for the influential factors in the extraction method had been established, they were used to determine the optimal extraction time of the same. For this purpose, several extractions were carried out where the optimal values of the factors already studied (percentage of methanol in the solvent, temperature, amplitude, cycle, pH, and sample–solvent ratio) remained invariable while different times were employed. The experiments were carried out in triplicate and the times studied were 2, 5, 10, 15, 20, 25, and 30 min. The results obtained are shown in Figure 5. Although each experiment has been carried out in triplicate, in the graph it has been represented as the mean value of the 3 measurements. It has been also included the error bars, which are graphical representations of the variability of data, i.e., the deviation standard (SD) of the 3 measurements. All these statistical analyses have been carried out with the software Microsoft Office Professional Plus 2016.

It can be seen from Figure 5 how the extracts that were subjected to ultrasound for 2 and 5 min produced greater yields, that is, a greater amount of total flavonols. On the other hand, longer extraction times (time > 5 min) led to lower recoveries. This could be explained by the degradation of the flavonols when they are subjected to ultrasounds for a long time at the set extraction temperature (60 °C). Therefore, a time a short as 2 min was determined as the optimum time for the extraction of flavonols from onion extract samples.

Finally, the precision of the developed UAE method was evaluated in terms of repeatability and intermediate precision. For this purpose, 10 experiments per day were conducted on 3 consecutive days to make a total of 30 experiments. Then, the intermediate precision was evaluated by analyzing the coefficient of variation (CV) of the 30 experimental results, while the repeatability was determined by calculating the coefficients of variation of each one of the 10 experiments performed on a single day. This method to determine a particular process precision has been typically employed in other works on similar natural matrices [18,42]. The percentage of repeatability obtained was 2.61%, while the intermediate precision was 3.05%. Since both coefficients of variation values are lower than 5%—the variation limit that is usually established for this type of processes—the method can be considered to have good repeatability and intermediate precision [43].

### 3.3. Development of Individual UAE Method for Antioxidant Activity

#### 3.3.1. Box–Behnken Design for Individual Antioxidant Activity

The large amount of flavonols that were quantified in UAE onion extracts lead us to think that this type of extract could be considered a rich source of phenolic compounds for its high content in quercetin derivatives and, therefore, it was expected to exhibit a good antioxidant capacity. Therefore, in order to evaluate the antioxidant activity of the onion extracts produced, as well as their correlation with the total flavonol content in the extracts, a second experimental design was elaborated. In this experimental design, the effect of the same 6 factors (percentage of methanol in the solvent, temperature, amplitude, cycle, pH, and ratio) as for the determination of flavonol contents, was studied, but this time, the target was to determine their effect on a new response variable: antioxidant activity (Y_DPPH_) as measured by DPPH assay. The same range of values were set for the six factors of interest. The results from this second experimental design (Table 1) were analyzed by ANOVA and the results obtained can be seen in Table 3.

Based on test-*F* (model *F*-value of 3.69), the *p*-value of the model was less than 0.05, which means a model significance at the 95% confidence level. However, as in the case of flavonols, the lack-of-fit test showed a *p*-value < 0.0001, which indicates evidence for a lack of fit, i.e., regression seems inadequate. In this case, as aforementioned, it is even more logical due to the large number of substances with different natures that can influence the antioxidant activity of the onion. Based on these results, it can be confirmed that the analysis explains 79.31% of the total variability.

**Figure 6:**
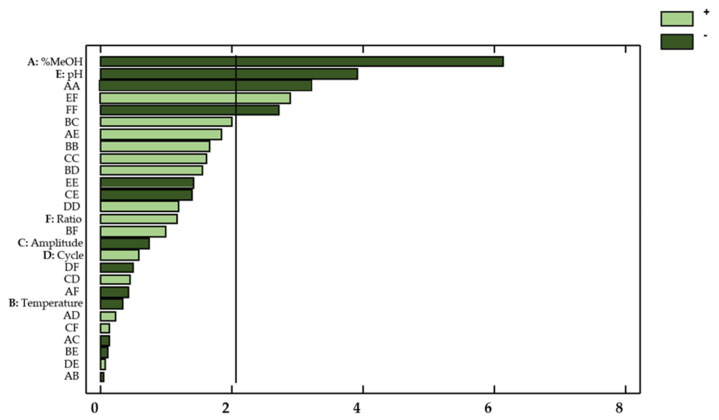
Standardized Pareto Chart representing the antioxidant activity of onion extracts. Light green (positive effect); Dark green (negative effect).

Thanks to the ANOVA and the Pareto chart (Figure 6), the factors or interactions that exhibited a remarkable influence on the antioxidant activity of the onion extracts could be determined.

In this regard, both linearly varying percentages of methanol in the solvent (X_1_) and pH levels (X_5_) showed a significant effect on the response (*p*-value < 0.05). With regard to the effect from interactions, the quadratic interaction percentage methanol–percentage methanol (X_1_^2^), the quadratic interaction ratio–ratio (X_6_^2^), and the interaction pH–ratio (*X_5_X_6_)* also showed a noticeable effect on the response.

Unlike what happened with flavonol content, higher percentages of methanol had a negative effect on the response variable (b_1_ = −1.37). pH also showed an inverse relationship with the extract antioxidant capacity (b_5_ = −0.87). These results indicate that a decrease in the solvent percentage of methanol and a decrease in the pH improve the antioxidant activity of the extracts. With respect to pH, the result obtained is logical, since, as mentioned previously, acidic pH favors the extraction of phenolic compounds due to play a key role in the breakdown of cell membranes. Therefore, if acidic pH in the solvent favors the extraction of the flavonols, it is logical that the antioxidant capacity of the extracts is also greater when using solvents with similar characteristics. As previously discussed, numerous articles have confirmed the linear relationship between polyphenolic content and antioxidant capacity [3,44,45]. With respect to the methanol in the solvent, an opposite trend is observed, which may be due to the fact that flavonols are not the only compounds responsible for the antioxidant capacity of onion extracts. This led us to assume that other compounds such as anthocyanins or phenolic acids have an important role in the said activity.

The polynomial equation (Equation (5)) for the antioxidant activity, obtained from the coefficients of the effects from the factors of interest and their interactions (Table 4) and the reduced equation (Equation (6)), obtained by omitting the irrelevant terms (*p*-valor > 0.05), were expressed as follows:

*Y_TF_*(mg g^−1^) = 9.50 − 1.37·*X_1_* − 0.073·*X_2_* − 0.16·*X_3_* + 0.13·*X_4_* − 0.87·*X_5_* + 0.26·*X_6_* − 1.10·*X_1_^2^* − 0.017 ·*X_1_X_2_* − 0.048·*X_1_X_3_* + 0.063·*X_1_X_4_* + 0.71·*X_1_X_5_* − 0.16·*X_1_X_6_* + 0.57·*X_2_^2^* + 0.77·*X_2_X_3_* + 0.59·*X_2_X_4_* − 0.028·*X_2_X_5_*+ 0.38·*X_2_X_6_* + 0.55·*X_3_^2^* + 0.17·*X_3_X_4_* − 0.53·*X_3_X_5_* + 0.037·*X_3_X_6_* + 0.41·*X_4_^2^* + 0.020·*X_4_X_5_* − 0.19·*X_4_X_6_* − 0.48·*X_5_^2^* + 1.11·*X_5_X_6_* − 0.93·*X_6_^2^*, (5)

*Y_TF_* (mg g^−1^) = 9.50 − 1.37·*X_1_* − 0.87·*X_5_* − 1.10·*X_1_^2^* + 1.11·*X_5_X_6_* − 0.93·*X_6_^2^*, (6)

**Figure 7:**
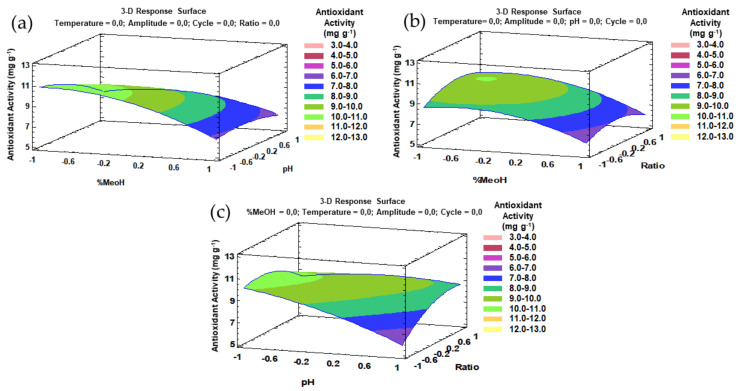
3D surface plots of the Box–Behnken design using polynomial equations: (**a**) solvent composition and pH on the antioxidant activity; (**b**) solvent composition and ratio on the antioxidant activity; (**c**) pH and ratio on the antioxidant activity.

Similarly to the phenolic compounds, for a better understanding of the main effects and the interaction effects from the most influential parameters, the trends that have been outlined above can be represented by three-dimensional (3D) surface plots based on the fitted model. The combined effects of the methanol–pH, methanol–ratio, and pH–ratio on the antioxidant activity of the onion extracts are represented in Figure 7a–c.

#### 3.3.2. Optimal Conditions for Individual Antioxidant Activity

The Box–Behnken design provided the necessary information with regard to the optimal values of each factor for a maximum response, that is, the best antioxidant activity. Specifically, for UAE, the following optimal values were obtained: 62.5% methanol in water with pH 2 as solvent, 57 °C extraction temperature, 90% ultrasound amplitude, 0.96 s ultrasound cycle, and 0.2:13.6 g:mL sample–solvent ratio. It can be seen that just like in the case of flavonols, high temperature and acid pH were established as optimal values. The optimal percentage of methanol in the solvent to favor antioxidant activities was lower, at 65.5% compared with the optimal percentage for the extraction of flavonols at 79%. This is probably due to the effect that the rest of the bioactive compounds, such as anthocyanins or phenolic acids, have on the extract’s antioxidant capacity.

It should also highlight the importance of the sample–solvent ratio as a key factor for the production of onion extracts with a high antioxidant capacity. The sample–solvent ratio is also a crucial factor with regards to production capacity since they have a direct influence on the cavitation phenomenon and on the final concentration of the extracts [46]. According to Chaves et al. [47], when the objective of the extraction is the preparation of samples for quantitative analysis, high solvent ratios (1:50, 1:100, or even higher) may be required to ensure that the target compounds are entirely removed from the sample matrix.

#### 3.3.3. Optimal Extraction Time and Precision for Individual Antioxidant Activity

**Figure 8:**
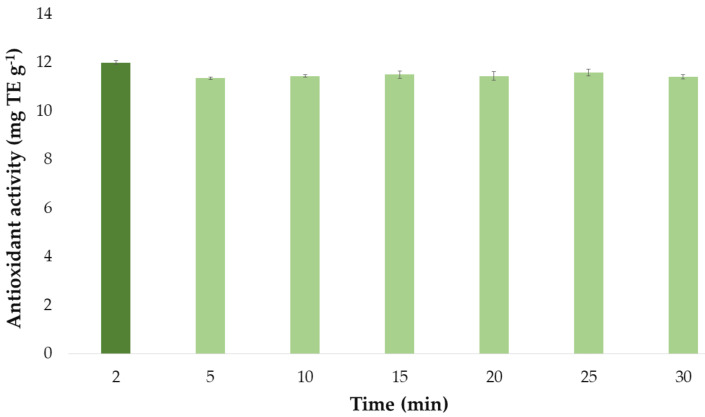
Optimal time (*n* = 3) for the maximum antioxidant activity of onion extracts according to the developed UAE method.

Once the optimal values of the extraction method influential factors had been established, they were used to determine the optimal extraction time of the extraction process. The antioxidant activity by the extracts obtained at different extraction times (2, 5, 10, 15, 20, 25, and 30 min) are presented in a bar graph (Figure 8) similar to the one used for the flavonoid extractions above. Each bar represented the mean value of the 3 measurements and it has also included the error bars, which are graphical representations of the variability of data, i.e., the deviation standard (SD) of the 3 measurements. All these statistical analyses have been carried out with the software Microsoft Office Professional Plus 2016.

It can be seen that the extracts that had been obtained by applying ultrasounds for just 2 min exhibited the greatest antioxidant activity. Again, this could be explained by the fact that bioactive compounds responsible for the antioxidant activity of onion extracts are degraded when exposed to ultrasound for a long time at the set extraction temperature (57 °C). For this reason, as for flavonoids, 2 min was selected as the optimum extraction time for maximum antioxidant activity.

As a final step to complete this study, the precision of the developed method was evaluated in terms of repeatability and intermediate precision. The repeatability coefficient obtained was 3.95%, while the intermediate precision coefficient was 4.84%. Both values are lower than 5%, so the method can be considered to have good repeatability and intermediate precision.

### 3.4. Development of a Simultaneous Extraction Method

Once both methods had been developed, a close similarity between the optimal values for most of the influential factors (Table 4) could be observed. Such a similarity seems to indicate that the antioxidant capacity of onions is mainly due to its flavonol content, which highlights the importance of quercetin derivatives. Quercetin derivatives are specific flavonols with very interesting antioxidant and free radical scavenging power [48] and onion is one of the natural sources with the highest content of these compounds. Thanks to the abovementioned similarity between the optimal values of the influential factors in both methods, they could be applied in combination to obtain flavonol-rich onion extracts with high antioxidant activity in a single process. Therefore, the analysis of their simultaneous responses is particularly interesting. For this purpose, multi-response optimization is a useful statistical tool that allows determining optimum balanced conditions to successfully obtain extracts with high antioxidant activity and high flavonol content. This is a particularly interesting aspect for quality control and analytical laboratories, where time and costs should be minimized [49]. The simultaneous optimal conditions to maximize both responses—flavonol content and antioxidant activity—are presented in Table 4.

When the multiple responses were optimized, extracts with high antioxidant activity (11.85 ± 0.11 mg TE g^−1^ DW) and high flavonol content (8.78 ± 0.03 mg flavonols g^−1^ DW) could be obtained in a single extraction (one extraction cycle optimized for both response factors). Although the results obtained when applying the optimal values resulting from the multi-response study were slightly lower than those obtained when the specific optimal values for flavonol extraction or antioxidant activities were applied, no significant differences were observed according to the *t*-test assuming equivalent variances (*p*-value < 0.05). Therefore, it can be affirmed that the optimized conditions for the simultaneous method can be applied instead of their specifically optimized extraction conditions with the subsequent saving in time and costs. The optimal time for the simultaneous method was set at 2 min since both individual methods showed the same optimal extraction times.

In the same way it was done for the separate extraction methods, the precision of the multi-response one was also evaluated. In terms of repeatability, the results obtained were: 2.45% for total flavonols and 3.04% for antioxidant activity, while for intermediate precision, the results were the following: 3.19% for total flavonols and 4.10% for antioxidant activity. Given that all the coefficients of variation were lower than 5%, the multi-response method’s precision was confirmed to be within acceptable limits.

Finally, and in order to demonstrate the advantages and interest of our study, the results that had been obtained were compared with those reported by other authors who had used the same natural matrix. Thus, our multi-response UAE method, which had been applied under optimal extraction conditions, achieved extraction of 8.78 ± 0.03 mg flavonols g^−1^ DW from red onion matrices, with most of it attributable to quercetin 3,4′-*O*-diglucoside and quercetin 4′ *O*-glucoside, since they represent about 90% of the overall flavonol contents. Subsequently, these results were compared to those reported by other authors who had used UAE, although no previous optimization of the influencing variables had been implemented. They do not carry out a full optimization of the variables that affect extraction. At this point, we should highlight how multiple variables affect extraction and how important it is to carry out its optimization to produce higher yields. In this sense, Corell et al. [50] did not carry out any optimization of the extraction variables. In the case of Jin et al. [51], even though they used a surface response methodology, the only factors to be optimized were solvent, temperature, ultrasound energy, and time. This partial optimization of the extraction factors resulted in longer extraction times (30 and 22 min respectively) compared to the optimal 2 min extraction time required by this method. Furthermore, the concentration of total flavonols in the resulting extracts were also lower (0.387 mg g^−1^ and 3.76 mg g^−1^, respectively). Other authors like Martino and Guyer [52], and Turner et al. [53], have used other types of advanced extraction techniques, such as supercritical fluids or subcritical water, respectively. However, they reported lower extraction yields from similar onion matrices in their studies (0.024 mg g^−1^ and 1.97 mg g^−1^), while the employed extraction times were substantially longer (2.5 h and 15 min).

With regard to the antioxidant capacity of the onion extracts obtained through our optimized multi-response UAE method, they exhibit levels as high as 11.85 ± 0.11 mg TE g^−1^ DW. Again, this was a much higher level than those reported by other authors who had also applied UAE to similar matrices. Aguiar et al. [2], in particular, declared an antioxidant activity of 1.042 mg TE g^−1^ of their onion extracts, while Gu et al. stated an antioxidant activity of their onion extracts at just 0.17 mg TE g^−1^.

Based on these results, it can be concluded that the multi-response UAE method that has been developed in this work allows higher extraction yields and shorter analysis time than any of the methods previously reported in the literature. Furthermore, the results from the antioxidant assay suggest a considerable radical scavenger activity of the resultant extract, what makes of it a very interesting food ingredient towards a healthier diet.

### 3.5. Application of the Developed Methods to Different Onion Varieties

Once the multi-response UAE method had been developed and optimized, it was applied to different onion varieties in order to verify its efficacy when applied to onions of different chemical composition. Specifically, 12 onion varieties were studied. The characteristics of the different onions in the study (their color, origin, producer, calibre, etc.) as well as a photography of each one of them can be seen in the Supplementary Material (Figure S1 and Table S1). The optimization of extraction method had been carried out using onion “12. Red onion IV”. The results from applying the developed multi-response UAE method to the different onion varieties are shown in Table 5. The results presented correspond to the mean of three replicates ± the standard deviation of these measurements.

According to these results, it can be concluded that the developed UAE method was applicable to the production of extracts from different onion varieties. This is of great interest, among other things, because it allows the characterization of onion varieties according to their flavonol and antioxidant profile. For example, according to these results, it can be also observed that the antioxidant activity is considerably higher in red onions (10.80 ± 0.47–12.65 ± 0.03 mg TE g^−1^ DW) than in white and yellow onions (7.82 ± 0.36–9.78 ± 0.41 mg TE g^−1^ DW). However, with respect to the flavonol content, this trend is less clear. Some specific white varieties, such as number 8 *Chalota onion* with 11.39 ± 0.21 mg flavonol g^−1^ DW, and number 9 *White onion* 14.08 ± 0.25 mg flavonol g^−1^ DW, have greater flavonol contents than the red varieties, with only 8.78 ± 0.03–10.56 ± 0.01 mg flavonol g^−1^ DW. This may be due to the effect that anthocyanins, compounds present mainly in red onions and almost absent in white onions [5], have on antioxidant capacity and that give the highest antioxidant capacity to the extracts of red onion varieties.

## 4. Conclusions

In this work, ultrasound-assisted extraction with BBD design has been used to obtain extracts with high antioxidant capacity and high flavonol content from red onion bulbs. Regarding the flavonols, the most influential extraction variables were: percentage of methanol (79% methanol in water), ultrasound amplitude (53.5%), and pH 2. Regarding the antioxidant activity, the most influential extraction variables were: percentage of methanol (79% methanol in water), pH 2, and solvent-sample ratio (0.2:13.6 g:mL). Both optimized extraction methods showed a very short extraction time (just 2 min), optimal extraction yields, and high repeatability and intermediate precision (RSD < 5%). Furthermore, due to the similarity in the optimal values for several variables, a simultaneous extraction method was developed employing the desirability function. In fact, this multi-response method produced 8.78 ± 0.03 mg flavonols g^−1^ DW with 11.85 ± 0.11 mg TE g^−1^ DW antioxidant activity, which means no significant differences (*p*-values < 0.05) with respect to each optimized individual extraction methods. Finally, the developed multi-response UAE method was successfully applied to many onion varieties to verify the applicability of the method to the production of extracts from onions with different chemical composition.

## Figures and Tables

**Table 1:** Experimental and predicted values for total flavonols and antioxidant activity based on the previously developed Box–Behnken design.

Run	Factors						Responses			
	X_1_	X_2_	X_3_	X_4_	X_5_	X_6_	Y_TF_ (mg Flavonols g^−1^ DW)		Y_DPPH_ (mg TE g^−1^ DW)	
							Experimental	Predicted	Experimental	Predicted
1	0	0	−1	0	−1	−1	8.00	7.94	10.41	10.02
2	0	0	1	0	−1	−1	7.59	7.78	10.41	10.69
3	0	0	−1	0	1	−1	7.15	7.03	9.80	7.12
4	0	0	1	0	1	−1	7.09	6.72	5.05	5.65
5	0	0	−1	0	−1	1	6.67	6.89	8.27	8.23
6	0	0	1	0	−1	1	6.65	6.92	6.93	9.05
7	0	0	−1	0	1	1	7.56	7.22	9.51	9.79
8	0	0	1	0	1	1	6.88	7.09	8.63	8.47
9	0	−1	0	−1	−1	0	7.25	7.26	11.00	11.37
10	0	1	0	−1	−1	0	7.33	7.57	10.68	10.09
11	0	−1	0	1	−1	0	6.90	7.13	11.11	10.41
12	0	1	0	1	−1	0	7.51	7.65	11.06	11.51
13	0	−1	0	−1	1	0	7.10	7.17	9.88	9.65
14	0	1	0	−1	1	0	7.24	6.79	7.77	8.26
15	0	−1	0	1	1	0	7.19	7.16	7.96	8.76
16	0	1	0	1	1	0	7.22	7.00	10.34	9.75
17	−1	0	−1	−1	0	0	2.96	3.47	10.60	10.92
18	1	0	−1	−1	0	0	6.88	6.55	8.31	8.16
19	−1	0	1	−1	0	0	3.16	3.39	10.44	10.35
20	1	0	1	−1	0	0	6.52	6.07	7.98	7.39
21	−1	0	−1	1	0	0	2.77	3.30	10.17	10.72
22	1	0	−1	1	0	0	6.82	6.52	8.08	8.21
23	−1	0	1	1	0	0	3.09	3.49	10.72	10.83
24	1	0	1	1	0	0	6.91	6.32	8.41	8.13
25	0	−1	−1	0	0	−1	7.36	7.38	10.89	10.84
26	0	1	−1	0	0	−1	7.25	7.52	5.96	8.40
27	0	−1	1	0	0	−1	6.74	7.14	8.75	8.91
28	0	1	1	0	0	−1	6.99	7.28	10.41	9.53
29	0	−1	−1	0	0	1	7.16	7.02	10.20	10.52
30	0	1	−1	0	0	1	7.27	7.02	10.32	9.60
31	0	−1	1	0	0	1	7.38	6.96	10.61	8.73
32	0	1	1	0	0	1	7.14	6.97	10.27	10.88
33	−1	−1	0	0	−1	0	4.65	4.37	11.82	11.44
34	1	−1	0	0	−1	0	6.80	6.59	6.86	7.33
35	−1	1	0	0	−1	0	5.68	4.88	12.51	11.38
36	1	1	0	0	−1	0	6.88	6.90	7.63	7.20
37	−1	−1	0	0	1	0	3.75	3.51	8.13	8.34
38	1	−1	0	0	1	0	6.81	7.39	6.14	7.06
39	−1	1	0	0	1	0	2.92	3.34	8.43	8.17
40	1	1	0	0	1	0	6.53	7.02	6.22	6.82
41	−1	0	0	−1	0	−1	5.56	4.86	8.37	8.55
42	1	0	0	−1	0	−1	6.60	7.07	5.71	6.02
43	−1	0	0	1	0	−1	4.15	4.14	8.64	9.07
44	1	0	0	1	0	−1	6.89	6.49	7.18	6.79
45	−1	0	0	−1	0	1	2.70	3.17	9.42	9.77
46	1	0	0	−1	0	1	6.79	6.72	6.97	6.59
47	−1	0	0	1	0	1	4.36	3.82	9.80	9.53
48	1	0	0	1	0	1	6.74	7.52	6.81	6.60
49	0	0	0	0	0	0	7.35	7.50	9.30	9.49
50	0	0	0	0	0	0	7.64	7.50	9.65	9.49
51	0	0	0	0	0	0	7.75	7.50	9.51	9.49
52	0	0	0	0	0	0	7.53	7.50	9.44	9.49
53	0	0	0	0	0	0	7.31	7.50	9.48	9.49
54	0	0	0	0	0	0	7.41	7.50	9.53	9.49

**Table 2:** Analysis of variance of the quadratic model adjusted for total flavonols in onion extracts.

Source	Source Code	Coefficients	Sum of Squares	Degrees of Freedom	Mean Square	*F*-Value	*p*-Value
Model		7.50	112.43	27	4.16	16.23	<0.001
A: %MeoH	*X_1_*	1.48	52.23	1	52.23	203.52	<0.001
B: Temperature	*X_2_*	0.036	0.030	1	0.030	0.12	0.734
C: Amplitude	*X_3_*	−0.072	0.12	1	0.12	0.48	0.494
D: Cycle	*X_4_*	0.019	0.0090	1	0.0090	0.04	0.853
E: pH	*X_5_*	−0.19	0.82	1	0.82	3.21	0.0849
F: Ratio	*X_6_*	−0.17	0.69	1	0.69	2.67	0.114
AA	*X_1_^2^*	−1.94	38.79	1	38.79	151.15	<0.001
AB	*X_1_X_2_*	−0.050	0.020	1	0.020	0.08	0.780
AC	*X_1_X_3_*	−0.10	0.079	1	0.079	0.31	0.584
AD	*X_1_X_4_*	0.037	0.022	1	0.022	0.08	0.774
AE	*X_1_X_5_*	0.41	1.37	1	1.37	5.35	0.0288
AF	*X_1_X_6_*	0.34	0.90	1	0.90	3.53	0.0717
BB	*X_2_^2^*	−0.046	0.022	1	0.022	0.09	0.772
BC	*X_2_X_3_*	<0.001	<0.001	1	<0.001	0.00	0.997
BD	*X_2_X_4_*	0.053	0.023	1	0.023	0.09	0.769
BE	*X_2_X_5_*	−0.17	0.47	1	0.47	1.84	0.187
BF	*X_2_X_6_*	−0.035	0.0097	1	0.0097	0.04	0.848
CC	*X_3_^2^*	−0.44	1.99	1	1.99	7.75	0.00990
CD	*X_3_X_4_*	0.070	0.039	1	0.039	0.15	0.699
CE	*X_3_X_5_*	−0.038	0.011	1	0.011	0.04	0.836
CF	*X_3_X_6_*	0.045	0.033	1	0.033	0.13	0.723
DD	*X_4_^2^*	−0.23	0.54	1	0.54	2.11	0.159
DE	*X_4_X_5_*	0.031	0.0075	1	0.0075	0.03	0.865
DF	*X_4_X_6_*	0.34	0.94	1	0.94	3.67	0.066
EE	*X_5_^2^*	−0.0082	<0.001	1	<0.001	0.00	0.960
EF	*X_5_X_6_*	0.31	0.76	1	0.76	2.96	0.0970
FF	*X_6_^2^*	−0.14	0.22	1	0.22	0.86	0.361
Residual			6.67	26	0.26		
Lack-of-Fit			6.52	21	0.31	10.55	0.0079
Pure Error			0.1472	5	0.029		
Cor Total			119.10	53			

**Table 3:** Analysis of variance of the quadratic model adjusted to the antioxidant activity of onion extracts.

Source	Source Code	Coefficients	Sum of Squares	Degrees of Freedom	Mean Square	*F*-Value	*p*-Value
Model		9.49	118.69	27	4.40	3.69	<0.001
A: %MeoH	*X*_1_	−1.37	44.79	1	44.79	37.61	<0.001
B: Temperature	*X*_2_	−0.073	0.13	1	0.13	0.11	0.747
C: Amplitude	*X*_3_	−0.16	0.64	1	0.64	0.54	0.470
D: Cycle	*X*_4_	0.13	0.42	1	0.42	0.35	0.559
E: pH	*X*_5_	−0.87	18.12	1	18.12	15.22	<0.001
F: Ratio	*X*_6_	0.26	1.60	1	1.60	1.34	0.258
AA	*X*_1_^2^	−1.10	12.53	1	12.53	10.52	0.00320
AB	*X*_1_ *X*_2_	−0.017	0.0023	1	0.0023	0.00	0.966
AC	*X*_1_ *X*_3_	−0.048	0.020	1	0.020	0.02	0.902
AD	*X*_1_ *X*_4_	0.063	0.063	1	0.063	0.05	0.820
AE	*X*_1_ *X*_5_	0.71	3.99	1	3.99	3.35	0.079
AF	*X*_1_ *X*_6_	−0.16	0.21	1	0.21	0.18	0.676
BB	*X*_2_^2^	0.57	3.31	1	3.31	2.78	0.108
BC	*X*_2_ *X*_3_	0.77	4.71	1	4.71	3.95	0.0575
BD	*X*_2_ *X*_4_	0.59	2.83	1	2.83	2.37	0.136
BE	*X*_2_ *X*_5_	−0.028	0.012	1	0.012	0.01	0.920
BF	*X*_2_ *X*_6_	0.38	1.16	1	1.16	0.98	0.332
CC	*X*_3_^2^	0.55	3.09	1	3.09	2.60	0.119
CD	*X*_3_ *X*_4_	0.17	0.24	1	0.24	0.20	0.660
CE	*X*_3_ *X*_5_	−0.53	2.29	1	2.29	1.92	0.178
CF	*X*_3_ *X*_6_	0.037	0.022	1	0.022	0.02	0.893
DD	*X*_4_^2^	0.41	1.69	1	1.69	1.42	0.244
DE	*X*_4_ *X*_5_	0.020	0.0033	1	0.0033	0.00	0.959
DF	*X*_4_ *X*_6_	−0.19	0.29	1	0.29	0.24	0.629
EE	*X*_5_^2^	−0.48	2.40	1	2.40	2.02	0.168
EF	*X*_5_ *X*_6_	1.11	9.93	1	9.93	8.34	0.00770
FF	*X*_6_^2^	−0.93	8.81	1	8.81	7.40	0.0115
Residual			30.9	26	1.19		
Lack-of-fit			30.90	21	1.47	111.66	<0.001
Pure Error			0.0659	5	0.0132		
Cor total			149.66	53			

**Table 4:** Individual and simultaneous optimal conditions to obtain extracts with high antioxidant activity as well as a large flavonol content.

Factor	Total Flavonols	Antioxidant Activity	Total Flavonols and Antioxidant Activity
% MeOH	79	62.5	76.9
Temperature (°C)	60	57	58.8
Amplitude (%)	53.5	90	85
Cycle (s)	0.54	0.96	0.94
pH	2	2	2
Ratio (g mL^−1^)	0.2:10.8	0.2:13.6	0.2:12.8
Result (mg g^−1^) ± SD (*n* = 3)	8.92 ± 0.02	12.00 ± 0.07	8.78 ± 0.03 and 11.85 ± 0.11

**Table 5:** Quantification of flavonols and antioxidant activity of the extracts (*n* = 3) from different onion varieties obtained by applying the developed multi-response UAE method.

Multiresponse UAE Method									
	Peak 1 ^a^	Peak 2 ^a^	Peak 3 ^a^	Peak 4 ^a^	Peak 5 ^a^	Peak 6 ^a^	Peak 7 ^a^	Total Flavonols ^b^	Antioxidant Activity ^b^
Units	mg Flavonols g^−1^ DW								mg TE g^−1^ DW
1. White onion I “spring”	0.16 ± 0.00	0.71 ± 0.00	2.39 ± 0.19	0.54 ± 0.00	0.58 ± 0.00	1.67 ± 0.14	0.41 ± 0.02	6.45 ± 0.35	9.74 ± 0.38
2. Red onion I	2.75 ± 0.01	0.84 ± 0.01	3.82 ± 0.47	0.65 ± 0.00	0.39 ± 0.01	2.08 ± 0.29	0.47 ± 0.02	9.40 ± 0.08	12.51 ± 0.04
3. White onion II “French”	1.19 ± 0.00	0.70 ± 0.00	2.76 ± 0.06	0.48 ± 0.00	1.01 ± 0.02	2.94 ± 0.07	0.33 ± 0.00	11.01 ± 0.51	9.37 ± 0.44
4. White onion III “sweet”	0.83 ± 0.00	0.60 ± 0.00	1.95 ± 0.06	0.46 ± 0.01	0.51 ± 0.00	2.05 ± 0.04	0.23 ± 0.01	1.89 ± 0.09	7.32 ± 0.17
5. Red onion II “Label”	0.29 ± 0.01	<LOQ	0.84 ± 0.02	<LOQ	0.44 ± 0.00	0.32 ± 0.00	<LOQ	10.56 ± 0.01	12.65 ± 0.03
6. Yellow onion	1.80 ± 0.08	0.83 ± 0.03	3.58 ± 0.22	0.75 ± 0.00	0.57 ± 0.00	2.09 ± 0.10	0.61 ± 0.02	6.63 ± 0.12	11.36 ± 0.17
7. Red onion III	1.05 ± 0.05	1.16 ± 0.02	4.08 ± 0.15	0.70 ± 0.01	0.58 ± 0.00	2.49 ± 0.09	0.49 ± 0.00	10.23 ± 0.21	10.80 ± 0.47
8. Chalota onion	<LOQ	0.65 ± 0.01	1.48 ± 0.07	0.44 ± 0.00	0.49 ± 0.00	1.01 ± 0.05	0.25 ± 0.00	11.39 ± 0.21	9.78 ± 0.41
9. White onion IV	2.66 ± 0.15	0.87 ± 0.00	4.78 ± 0.03	0.62 ± 0.01	0.91 ± 0.00	3.67 ± 0.02	0.57 ± 0.01	14.08 ± 0.25	9.38 ± 0.43
10. White onion V “sweet”	<LOQ	<LOQ	0.71 ± 0.13	<LOQ	3.20 ± 0.12	0.12 ± 0.01	<LOQ	2.64 ± 0.14	7.82 ± 0.36
11. White onion VI “spring”	1.77 ± 0.16	1.22 ± 0.01	4.11 ± 0.24	0.85 ± 0.00	0.63 ± 0.00	2.19 ± 0.16	0.61 ± 0.02	4.22 ± 0.05	6.85 ± 0.08
12. Red onion IV	0.94 ± 0.09	0.77 ± 0.00	2.46 ± 0.02	0.81 ± 0.01	0.58 ± 0.00	2.43 ± 0.01	0.79 ± 0.00	8.78 ± 0.03	11.85 ± 0.11

^a^ Peak 1. quercetin 3,7,4′-O-triglucoside, peak 2. quercetin 7,4′-O-diglucoside, peak 3. quercetin 3,4′-O-diglucoside, peak 4. isorhamnetin 3,4′-O-diglucoside, peak 5. quercetin-O-glucoside, peak 6. quercetin 4′-O-glucoside, peak 7. isorhamnetin 4′-O-glucoside. ^b^ Flavonols composition and antioxidant activity expressed as mean of three replicates ± standard deviation. <LOQ = Below the limit of quantification.

## Data Availability

The data presented in this study is contained within the article or supplementary material.

## Supplementary Materials

The following are available online at https://www.mdpi.com/2076-3921/10/2/273/s1, Figure S1: Photograph of each variety of onions studied, Table S1: Characteristics of the onions studied.
