# Supplementary material for: Flavonol Composition and Antioxidant Activity of Onions (Allium cepa L.) Based on the Development of New Analytical Ultrasound-Assisted Extraction Methods
Source: Antioxidants (Basel). 2021 Feb 10;10(2):273. doi: 10.3390/antiox10020273 (PMC7916630; doi:10.3390/antiox10020273)
Supplement: Supplementary file 1 [file antioxidants-10-00273-s001.pdf]

Flavonol Composition and Antioxidant Activity of Onions (*Allium cepa* L.) Based on the Development of New Analytical Ultrasound-Assisted Extraction Methods

Ana V. González-de-Peredo, Mercedes Vázquez-Espinosa, Estrella Espada-Bellido, Ceferino Carrera, Marta Ferreiro-González, Gerardo F. Barbero * and Miguel Palma

Department of Analytical Chemistry, Faculty of Sciences, University of Cadiz, Agrifood Campus of International Excellence (ceiA3), IVAGRO, 11510, Puerto Real, Cadiz, Spain; ana.velascogope@uca.es (A.V.G.-d.-P.); mercedes.vazquez@uca.es (M.V.-E.); estrella.espada@uca.es (E.E.-B.); marta.ferreiro@uca.es (M.F.-G.); ceferino.carrera@uca.es (C.C.); gerardo.fernandez@uca.es (G.F.B.); miguel.palma@uca.es (M.P.)

* Correspondence: gerardo.fernandez@uca.es; Tel.: +34-956-01-6355

SUPPLEMENTARY MATERIAL

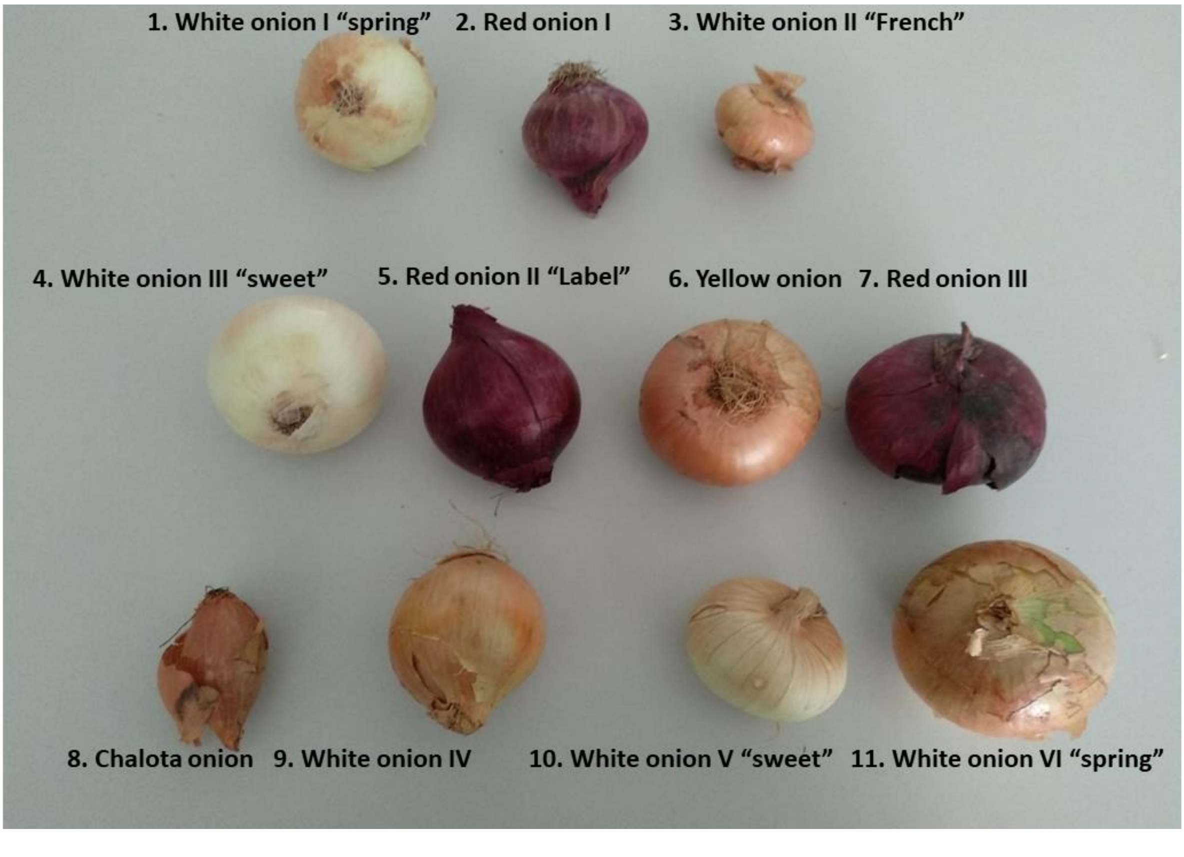

Figure S1. Photograph of each variety of onions studied.

Table S1. Characteristics of the onions studied.

Onion variety	Color	Origin	Company	Caliber
1. White onion I "spring"	White	Spain	La Gramola	50/90 mm
2. Red onion I	Red	Spain	La Gramola	50/70 mm
3. White onion II "French"	White	Spain	La Gramola	37/55 mm
4. White onion III "sweet"	White	Chile	-	-
5. Red onion II "Label"	Red	Spain	Linda	50/70 mm
6. Yellow onion	Yellow	Spain	-	-
7. Red onion III	Red	Spain	Tara	-
8. Chalota onion	White	French	Tara	24 mm
9. White onion IV	White	New Zealand	Tara	55 mm
10. White onion V "sweet"	White	Spain	Tara	60/90 mm
11. White onion VI "spring"	White	Spain	Tara	65 mm
12. Red onion IV	Red	Austria	Tara	50/90 mm
